# Obesity impairs academic attainment in adolescence: findings from ALSPAC, a UK cohort

**DOI:** 10.1038/ijo.2014.40

**Published:** 2014-04-08

**Authors:** J N Booth, P D Tomporowski, J M E Boyle, A R Ness, C Joinson, S D Leary, J J Reilly

**Affiliations:** 1 School of Psychology, University of Dundee, Dundee, UK; 2 University of Georgia, Athens, GA, USA; 3 University of Strathclyde, Glasgow, UK; 4 University of Bristol, Bristol, UK

**Keywords:** ALSPAC, academic attainment, adolescents

## Abstract

**Objective:**

While being overweight or obese in adolescence may have detrimental effects on academic attainment, the evidence base is limited by reliance on cross-sectional studies with small sample sizes, failure to take account of confounders and lack of consideration of potential mediators. The present study aimed to address these limitations and examine longitudinal associations between obesity in adolescence and academic attainment.

**Design:**

Associations between weight status at 11 years old and academic attainment assessed by national tests at 11, 13 and 16 years were examined in the Avon Longitudinal Study of Parents and Children. Healthy weight was defined as body mass index (BMI) *Z*-score <1.04; overweight as BMI *Z*-score 1.04–1.63; obesity as BMI *Z*-score ⩾1.64.

**Participants:**

Data from 5966 participants with objectively measured weight status were examined: 71.4% were healthy weight (1935 males; 2325 females), 13.3% overweight (372 males; 420 females) and 15.3% obese (448 males; 466 females).

**Results:**

Girls obese at 11 years had lower academic attainment at 11, 13 and 16 years compared with those of a healthy weight, even after controlling for a wide range of confounders. Associations between obesity and academic attainment were less clear in boys. The potential mediating effects of depressive symptoms, intelligence quotient (IQ) and age of menarche in girls were explored, but when confounders were included, there was no strong evidence for mediation.

**Conclusions:**

For girls, obesity in adolescence has a detrimental impact on academic attainment 5 years later. Mental health, IQ and age of menarche did not mediate this relationship, suggesting that further work is required to understand the underlying mechanisms. Parents, education and public health policy makers should consider the wide reaching detrimental impact of obesity on educational outcomes in this age group.

## Introduction

Systematic reviews on the impact of child and adolescent obesity have reported a wide variety of adverse consequences, in both the short and long term.^1,2^ Some evidence for a long-term adverse impact of childhood obesity on adult educational attainment and adult income was reported in the earliest of the systematic reviews on the consequences of childhood obesity; however, only two older studies were identified, one from the United States and the other from the United Kingdom.^1^ More recently, a systematic review by Caird *et al.*^3^ in 2011 included papers examining the hypothesis that child or adolescent obesity impairs academic attainment. While 29 eligible studies were included in this systematic review,^3^ almost all were based on samples from the United States and so not internationally representative. Most had serious limitations, notably the cross-sectional design, lack of control for socioeconomic status, an important confounder that increases risk of both poor educational attainment and obesity, and small sample size. Moreover, the review by Caird *et al.*^3^ noted that few studies had addressed the potential mediators of any relationships between child or adolescent obesity and academic attainment.

Since the publication of the review by Caird *et al.*,^3^ few robust studies which address the weaknesses identified in the evidence base have been published. Two analyses from the large UK Avon Longitudinal Study of Parents and Children (ALSPAC) cohort study have produced equivocal and apparently contradictory results from children and adolescents in the cohort.^4,5^ Cross-sectional analyses of the relationship between fat mass and attainment at 7 years old reported weak positive associations;^4^ however, further analyses found a negative relationship between fat mass at age 9 years and attainment at age 11 years, although genetic instrumental variable estimates suggested that the relationship was because of unobserved variables.^5^ These contradictory results may be related to the differing age groups and differences in choice of confounding variables. There is therefore a need to test whether, and to what extent, paediatric obesity impacts independently on subsequent academic attainment.

Empirical evidence of a number of potentially plausible mechanisms has come to light recently, including: adverse effects of obesity on physical and mental health (e.g. depressive symptoms) leading to absenteeism;^6^ ‘indirect mechanisms' such as possible stigmatisation by peers or teachers;^7^ more ‘direct' cognitive mechanisms by which excess adiposity might impair cognition (e.g. intelligence quotient (IQ)) and have adverse effects on learning and academic attainment;^8^ potential impact of early puberty in females that has been linked to both obesity and poor school performance.^9,10^ A recent review by Smith *et al.*^11^suggested that obesity is negatively associated with cognition in children, adolescents and adults, providing one plausible mechanism. However, some limitations of this recent evidence are also apparent, for example, dependence on self-reported measures of both exposures (weight status) and outcomes (academic grades) in some studies:^7^ the existence and relative importance of these various potential mechanisms therefore remains uncertain.

The primary aim of the present study was to test the hypothesis that adolescent obesity independently influences subsequent academic attainment by using a large, longitudinal UK study of adolescents, the ALSPAC.^12^ Secondary aims were to examine candidate underlying mechanisms in the relationship between adolescent obesity and subsequent academic attainment.

## Materials and methods

### Study cohort

The sample comprised participants from the ALSPAC.^13^ ALSPAC is an ongoing population-based study investigating influences on health and development of children. Ethical approval for the study was obtained from the ALSPAC Law and Ethics Committee and the Local Research Ethics Committees. Pregnant women resident in the former Avon Health Authority in south-west England, having an estimated date of delivery between 1 April 1991 and 31 December 1992, were invited to take part, resulting in a cohort of 14 541 pregnancies and 13 988 children alive at 12 months. When the oldest children were aged 7 years, an attempt was made to increase the size of the initial sample with eligible cases that did not join the cohort at the outset. The phases of enrolment are described in more detail in the cohort profile paper.^12^ Please note that the study website contains details of all the data that are available through a fully searchable data dictionary (http://www.bristol.ac.uk/alspac/researchers/data-access/data-dictionary/).

### Study design

The present longitudinal study is based on associations between objectively measured weight status at an ALSPAC research clinic attended at 11 years old and academic attainment at 11, 13 and 16 years old.

### Exposure, outcomes and covariates

#### Weight status

Weight status was defined on the basis of the body mass index (BMI) for age relative to UK 1990 population reference data, using research clinic measures of weight and height made at age 11 and 16 years. Healthy weight was defined as BMI *Z*-score <1.04; overweight as BMI *Z*-score 1.04–1.63; obesity as BMI *Z*-score ⩾1.64.^14, 15, 16, 17^

#### Academic attainment

Academic attainment assessed at 11, 13 and 16 years old were the outcome measures for the present analyses. Compulsory national achievement tests were completed in England at age 6/7 (Key Stage 1), age 10/11 (Key Stage 2), age 13/14 (Key Stage 3) and age 15/16 years (Key Stage 4: General Certificate of Secondary Education (GCSEs)). Data linkage with the National Pupil Database in England^18^ provided results of assessments in English, Maths and Science at Key Stages 2–4 (see Department of Education for further details).^19^ At Key Stages 2 and 3, raw scores are converted to levels (level 1–8, with 8 being the highest), and at Key Stage 4, GCSE's are graded from A* to G and U (ungraded), with A* being the highest. At age 11 and 13 years, raw scores for academic attainment were used in analyses, and at age 16 years, GCSE grades were converted from alphabetic grades (U to A*) to numerical values ranging from 1 to 9, with 9 being the highest.

#### Confounders

Potential confounders identified in previous literature were included in the analyses owing to their relationship with the exposures and outcome measures: age; birth weight; gestation; age of mother at delivery; mother's oily fish intake during pregnancy, as assessed by questionnaire at 32 weeks gestation; maternal smoking in the first 3 months of pregnancy; pubertal status based on Tanner pubic hair stage for males (stage I (least advanced) to V (most advanced)) and menarche status for females evaluated at age of outcome;^20, 21, 22^ ethnicity; socioeconomic status based on maternal educational attainment (none/Certificate of Standard Education to University degree) and occupational social class as classified by the Office of Population Censuses and Surveys in 1991 (classes I (professional/managerial) to V (unskilled manual workers));^23^ average daily number of minutes spent in moderate-to-vigorous physical activity (MVPA) at 11 years old assessed by accelerometry (procedure reported previously^24,25^); depressive symptoms at 11 years old reported by parents using the Short Moods and Feelings Questionnaire;^26,27^ and full-scale IQ estimated at 8 years old using the Wechsler Intelligence scale for children (WISC-III^UK^).^28^ In addition, for analyses of academic attainment at 16 years old, BMI *Z*-score at age 16 years was included as a further confounding variable.

### Inclusions and exclusions

The following criteria were used to exclude participants based on potential confounding of academic performance: a psychiatric diagnosis based on the Development and Well-being Assessment,^29^ which provides information to make a Diagnostic and Statistical Manual of Mental Disorders, Fourth Edition^30^ clinical diagnosis^31^ (*n*=277); participants with a Statement of Educational Needs as reported by school or parents (*n*=744); participants with Total Difficulties scores of 16 and greater on the teacher-completed version of the Strengths and Difficulties Questionnaire^32^ at age 11 years (*n*=228). Of this group of excluded participants, 20% were categorised as obese and 14.3% as overweight.

### Statistical analyses

The associations between weight status (healthy weight, overweight or obese) and academic attainment were assessed using linear regression analyses with dummy variables for weight status entered as predictor variables with healthy weight as the reference group. As males and females have been found to differ in relation to academic attainment,^33^ the interaction between sex and weight status was formally tested. There was evidence for interaction effects (*P*-values <0.05); therefore, analyses were conducted separately for males and females.

A series of models were used to explore the impact of confounding variables. Model 1 (minimally adjusted model) was unadjusted for any confounding variables. Model 2 adjusted for age of participants. Model 3 adjusted for the potential confounders in model 2, plus birth weight and gestational age. In model 4, the variables included in model 3 were adjusted for, together with age of mother at delivery, mother's oily fish intake and whether the mother of participants smoked in the first three months of pregnancy. Model 5 adjusted for potential confounding variables in model 4 plus the inclusion of pubertal stage of participant (recorded at time of outcome). Model 6 adjusted for all confounders in model 5 plus ethnicity, maternal educational attainment and occupational social class. Model 7 adjusted for all confounders in model 6 and also adjusted for the average daily number of minutes spent in MVPA. In model 8, all variables in model 7 were included with the addition of parent reported depressive symptoms at age 11 years. A fully adjusted model (model 9) controlled for all of the previous potential confounders plus full-scale IQ. An additional model (model 10) was also estimated for the relationship between weight status and academic attainment at 16 years old only. This model included all of the previously included confounders but also adjusted for BMI *Z*-score relative to UK 1990 reference data at 16 years old.

To test whether depressive symptoms and IQ mediate the relationship between weight status and academic attainment, a series of mediation models were performed. Furthermore, the potential mediating impact that age of menarche in girls had was also examined. Bootstrapped estimates of the indirect effects and associated 95% confidence intervals were calculated using the procedure described in Preacher and Hayes^34^ and using the macro provided for SPSS (Armonk, NY, USA).

To investigate whether change in weight status between 11 and 16 years old had an impact on academic attainment at age 16, dummy variables representing change status (stable healthy weight; stable overweight/obese; became overweight from healthy weight; became obese from healthy weight; became obese from overweight; became healthy weight from overweight/obese) were calculated with stable healthy weight as the reference group and entered as predictor variables in regression analyses.

SPSS version 19 was used for all analyses with list-wise deletion for regression models. To assess whether changes in effect sizes identified in models 2–9 were because of bias relating to missing data or not, model 1 was repeated for only those participants who had complete data in model 9 (complete confounder information).

## Results

### Characteristics of study participants

Of the 11 952 invited to attend the 11-year clinic, 60% attended, and objective measurements of height and weight were taken from 7095 of these participants. Following the application of all exclusion criteria, data from 5966 participants remained for analyses. In all, 71.4% of the samples (*n*=4260) were classed as healthy weight (1935 males; 2325 females), 13.3% (*n*=792) were classed as overweight (372 males; 420 females) and 15.3% (*n*=914) were classed as obese (448 males; 466 females). Table 1 provides the characteristics of these participants.

Participants were further grouped based on change in their weight status between 11 and 16 years old. Of the participants , 67.7% (*n*=2328) were classed as stable healthy weight (1026 males; 1302 females); 15.8% (*n*=543) were stable overweight/obese (255 males; 288 females); 4.8% (*n*=165) became overweight from healthy weight (58 males; 107 females); 1.2% (*n*=40) became obese from healthy weight (14 males; 26 females); 2.2% (*n*=77) became obese from overweight (25 males; 52 females); 8.3% (*n*=286) became healthy weight from being overweight/obese (151 males; 135 females).

As reported elsewhere,^21,24,25^ when comparisons of characteristics were made between those who attended the clinic and those who did not, small differences were found in birth weight, social class, maternal education, maternal height and age. Descriptive statistics for academic attainment are shown in Table 2.

### Associations with academic attainment for males

Linear regression analyses were used to examine associations between weight status at 11 years old and academic attainment in males at age 11, 13 and 16 years and the resulting *β*-coefficients for the unadjusted model and the fully adjusted model can be found in Table 3 (with results for each step of adjustment found in Supplementary Material). Results presented in Table 3 are for English attainment only, with results for Maths and Science given in Supplementary Material.

In the unadjusted model at age 11 years, participants in the obese group had lower English attainment than those in the healthy weight group. With adjustment for the full range of confounders, this continued to be the case with only small attenuation of the coefficient. While a similar pattern was found when weight status at 11 predicted academic attainment at 13 and 16 years, the attenuation with the addition of confounders was substantially greater and the resulting confidence intervals straddled zero, suggesting that there were no group differences in attainment. Similar patterns were found for Maths and Science, with obese participants generally having lower attainment than those of a healthy weight in the unadjusted models, but the associations attenuating with the addition of confounders. The resulting coefficients can be found in Supplementary Material.

A similar trend was found for comparison of those who were overweight with those of a healthy weight; however, the *β*-coefficients were small with wide confidence intervals, suggesting that weight status did not predict distinguishable differences in academic attainment for these groups.

### Associations with academic attainment for females

Table 4 reveals the *β*-coefficients for linear regression analyses in females with weight status at 11 years old predicting English attainment at 11, 13 and 16 years old. Results for the minimally adjusted model and the fully adjusted model only are shown in Table 4, with results for each step of adjustment shown in Supplementary Material.

The *β-*coefficients reveal that girls who were obese at age 11 years had lower academic attainment at age 11, 13 and 16 years than those of a healthy weight. Adjustment for the full range of confounders in the model at age 11 years resulted in attenuation of the coefficient and narrowing of confidence intervals. However, for association with attainment at age 13 and 16 years, the attenuation with the addition of confounders was smaller, suggesting that there were group differences over the long term. A further adjustment was made for attainment at 16 years, by adjusting for current weight (i.e. BMI *Z*-score at 16 years old) in the analyses (model 10). This led to a slight widening of the confidence intervals, but the resulting coefficient was not altered. This suggests that even when current weight status was controlled for, girls who were obese at age 11 years had lower academic attainment at 16 years old than those of a healthy weight.

A similar trend was found for comparison of those who were overweight with those of a healthy weight, with the unadjusted models suggesting poorer academic attainment at age 11, 13 and 16 years for those who were overweight at age 11 years. However, adjustment for confounders attenuated the coefficients and led to considerably wider confidence intervals, suggesting that when a wide number of confounders were taken into account there were no group differences.

Results for Maths and Science attainment showed more variation than English; however, there was a trend for a similar pattern of results at all three time points and the resulting coefficients can be found in Supplementary Material.

### Mediation analyses

A series of mediation analyses were performed to assess whether depressive symptoms or IQ mediated the relationship between weight status and academic attainment at 16 years old (see Supplementary Material for illustration). Confounding variables from the fully adjusted model were included in the analyses; therefore, the results show the extent to which depressive symptoms and IQ mediate the relationship between weight status and academic attainment at 16 years old, while taking into account the full range of confounding variables. Table 5 shows the total effects, direct effects and indirect effects for these analyses for overweight and obese participants compared with those of a healthy weight. The estimates and associated confidence intervals for the indirect effects demonstrate that neither depressive symptoms nor IQ mediated the relationship between weight status at 11 years and academic attainment at 16 years old for males or females.

The age at which girls experienced menarche was also examined as a potential mediator. Fourteen participants for whom this information was available at age 16 years had not yet experienced menarche and were therefore excluded. The resulting coefficients can be found in Table 5 and demonstrate that age at which girls experienced menarche did not mediate the relationship between weight status at 11 years old and academic attainment at 16 years.

### Change in weight between 11 and 16 years old

Associations between change in weight status between 11 and 16 years old and English attainment at 16 years old (GCSE grades) are shown in Table 6. For males, change in weight status did not predict academic attainment at 16 years old. However, females who were stable overweight/obese had lower attainment at 16 years old than those who were of a stable healthy weight even when controlling for a wide range of confounding variables. Furthermore, females who gained weight and went from being overweight at 11 years to obese at age 16 years also had a lower attainment than those of a stable healthy weight, even in the fully adjusted model. No meaningful associations were found for participants who were a healthy weight at 11 years but gained weight, or those who lost weight when compared with those who were of a stable healthy weight.

To assess whether changes in effect sizes identified in models 2–9 were due to bias because of missing data or not for each association, model 1 was repeated for participants who had complete data at model 9. The resulting coefficients were slightly larger than when all available data were included and results were summarised in Supplementary Material.

## Discussion

### Main findings and study implications

The present study suggests that adolescent obesity in the United Kingdom in the 2000s had an adverse impact on subsequent academic attainment in girls. Moreover, this adverse impact was robust to confounding variables, and was almost certainly of practical significance as it was sufficient to cross-grade boundaries in attainment. That is, for females, after controlling for a wide range of confounders, being obese at 11 predicted lower attainment by one-third of a grade at age 16 years. In the present sample, this would be sufficient to lower average attainment to a grade D instead of a grade C. Furthermore, examination of change in weight status found that females who were overweight or obese over the long term had lower attainment than those who were of a stable healthy weight. Being overweight or obese at age 16 years was not as detrimental for attainment if participants had been a healthy weight at age 11 years, suggesting that, potentially, it is long-term overweight/obesity that is the most problematic. Further work is needed to substantiate this though.

The present study therefore provides, possibly for the first time given the limitations of many previous studies,^3,35^ a clear support for the hypothesis that obesity is independently associated with poorer academic outcomes in adolescence. This result was robust in females, and while there was a trend for similar results in males, they were not as convincing. A small number of previous studies have reported stronger negative associations between BMI and cognition for females during childhood,^36,37^ suggesting that our finding of gender differences in associations is not necessarily spurious.

Impairments in academic outcomes in high school observed in the present study may at least partly explain decrements in adult educational attainment and income associated with adolescent obesity found in two older studies from the United States and United Kingdom.^38,39^ The association between obesity and impaired academic attainment may be explained by a range of plausible impacts of adolescent obesity on: physical and mental ill health and consequent absenteeism from school;^6^ ‘indirect effects' on teacher grades;^7^ evidence is also emerging for more direct mechanisms linking excess child or adolescent adiposity and/or the lifestyles associated with it, to impaired cognition.^8,35,39, 40, 41^ Studies using genetic markers have begun to emerge in the literature, and while some evidence supports the findings of stronger associations of obesity and academic attainment in females as in the present study,^42^ not all studies are consistent.

### Comparisons with other evidence

The recent systematic review of associations between obesity and academic attainment found that much of the previous evidence in this area was somewhat limited,^3^ in particular by the preponderance of cross-sectional studies, studies that did not or could not adjust for the confounding influence of socioeconomic status, and studies from one single setting (the United States). Nonetheless, the balance of previous evidence suggested that child and/or adolescent obesity might be associated with poorer academic attainment.^3^

The present study found that obesity was independently associated with academic attainment even after controlling for depressive symptoms, IQ and age of menarche in females, which are often purported to account for associations observed in other studies.^7^ None of these factors mediated the relationship though adding support to the proposition of causal relationships, although other candidate mediators should be explored in further research (see limitations). We are not aware of any studies to date which have attempted to explain associations between obesity and academic outcomes formally, using mediation analysis as in the present study.

### Study strengths and limitations

The present study had a number of important advantages over previous evidence;^3,35^ notably: relatively large sample size; longitudinal design; ability to adjust for a wide range of known confounders; good measures of exposures and outcomes; independent measures of academic outcomes with substantial practical significance (external public examination results at age 16 years); ability to examine both outcomes and potential mediators of the associations; and importantly, the ability to consider the possible impact of obesity as distinct from overweight. However, there remains some debate as to the causality of obesity–academic outcome relationships.^11,43^ Intervention studies might provide useful evidence, but most interventions that aim to improve weight status in obese children and adolescents report relatively small-modest effects,^44^ and in any event, an ongoing Cochrane review has found a dearth of such studies to date.^45^

The present study also had a number of limitations. Exclusion of participants with developmental difficulties allows us to generalise our findings typically to developing young people; however, it precludes us from making any observations about the groups of young people excluded from the present analyses. Differences in the nature of associations may be observed for individuals with developmental difficulties and so these groups are worthy of further investigation. While three potential mediators of the associations between obesity and academic outcomes were examined, not all candidate mediators were available for examination. For example, we had no measure of self-esteem or absenteeism^6^ or indeed the school environment and role of the teacher, which may also influence the relationships observed. The evaluation of a wider range of potential mediators would increase our understanding of this relationship. While we were able to adjust for a wide range of confounding variables, we were not in a position to evaluate whether change in any of the confounders (e.g. change in depressive symptoms) had an impact on the present findings. Furthermore, caution must be taken when interpreting the data owing to the large number of comparisons. In addition, for some variables the measure that was available in the ALSPAC cohort study was not the ideal measure of the candidate mediator. For example, components of executive functions may be important cognitive mediators,^11,39^ but the available measure of EF was not optimal for assessing the full range of potentially relevant cognitive pathways. Further research that provides a more comprehensive understanding of the underlying cognitive mechanisms based on measures of inhibition, working memory and shifting, for example, is required.

The loss of data in the fully adjusted models compared with the unadjusted models could be considered a limitation; however, no substantial differences were detected when models were reanalysed, including participants with complete confounding information only.

## Conclusions

This study suggests that obesity in adolescence is associated with poorer subsequent academic attainment for females, and that the magnitude of the relationship may be considered important. Moreover, the present study suggests that the relationship between obesity and subsequent academic attainment is likely to be causal, and provides evidence against some candidate mediators. Academic outcomes are of high importance to adolescents, parents, schools, school systems and to society,^40^ especially given the relationship between attainment and unemployment in young people.^46,47^ An adverse effect of obesity on academic outcomes might provide stakeholders with greater motivation to engage with initiatives to both treat and prevent paediatric obesity in future.

## Figures and Tables

**Table 1:** Characteristics of participants categorised by weight status at 11 years old

*Characteristic*	*Healthy weight*		*Overweight*		*Obese*	
	n	*Mean (s.d.)*	n	*Mean (s.d.)*	n	*Mean (s.d.)*
Age in months at weight status measurement	4259	140.94 (2.80)	792	140.98 (3.04)	914	141.03 (2.89)
Birth weight (g)	3987	3387.61 (526.66)	740	3475.29 (545.34)	842	3494.75 (570.38)
Gestation (weeks)	4043	39.45 (1.80)	746	39.49 (1.86)	850	39.45 (1.75)
Age of mother in years at delivery	4043	29.12 (4.54)	746	28.76 (4.54)	850	28.82 (4.63)
Average daily minutes of MVPA	3611	24 (16)	669	20 (14)	740	19 (13)
Depressive symptoms at 11 years	3569	1.99 (2.76)	651	2.11 (2.84)	715	2.49 (3.36)
Full-scale IQ at 8 years	3643	107.02 (15.34)	657	106.36 (15.64)	746	103.42 (16.39)
BMI *Z*-score at 11 years	4259	−0.25 (0.81)	792	1.32 (0.19)	914	2.19 (0.42)
BMI *Z*-score at 13 years	1827	−0.21 (0.96)	333	1.07 (0.56)	313	1.89 (0.64)
BMI *Z*-score at 16 years	2533	−0.07 (0.84)	443	1.00 (0.68)	463	1.82 (0.77)
						
		*Percentage*		*Percentage*		*Percentage*
*Proportion of sample*						
Female	4260	71.4	792	13.3	914	15.3
Ethnicity	2325	54.6	420	53.0	466	51.0
White	3716	96.4	672	95.6	753	94.6
Non-white	137	3.6	31	4.4	43	5.4
*Mother's oily fish intake*						
Never/rarely	1362	35.6	299	42.1	347	44.1
Once in 2 weeks	1373	35.9	243	34.2	253	32.2
1–3 times a week	1044	27.3	161	22.7	181	23.0
4–7 times a week	47	1.2	7	1.0	4	0.5
More than once a day	1	0.0	0	0.0	1	0.1
*Mother smoked during pregnancy*						
Yes	602	15.2	133	18.2	177	21.3
No	3369	84.8	599	81.8	654	78.7
*Mothers education*						
CBSE	462	11.8	103	14.3	142	17.7
Vocational	313	8.0	64	8.9	81	10.1
O level	1381	35.3	270	37.5	304	37.8
A level	1051	26.9	186	25.8	208	25.9
Degree	703	18.0	97	13.5	69	8.6
*Occupational social class*						
I (professional)	280	8.2	29	4.6	29	4.4
II	1192	34.9	207	33.2	225	34.3
III (non-manual)	1431	41.8	280	44.9	293	44.7
III (manual)	214	6.3	47	7.5	44	6.7
IV	265	7.7	53	8.5	53	8.1
V (unskilled)	37	1.1	8	1.3	12	1.8
Armed forces	1	0.0	0	0	0	0
*Pubertal status at 11 years*						
Experienced menarche	201	11.3	78	25.08	101	29.79
Tanner stage I	508	40.7	69	30.0	88	31.5
Tanner stage II	485	38.9	100	43.5	108	38.7
Tanner stage III	158	12.7	40	17.4	55	19.7
Tanner stage IV	42	3.4	13	5.7	13	4.7
Tanner stage V	2	0.2	13	5.7	1	0.4

Abbreviations: CBSE, Central Board of Secondary Education; IQ, intelligence quotient; MVPA, moderate-to-vigorous physical activity.

**Table 2:** Descriptive statistics for academic attainment

*Academic attainment*	*Healthy weight*				*Overweight*				*Obese*			
	*Males*		*Females*		*Males*		*Females*		*Males*		*Females*	
	n	*Mean (s.d.)*	n	*Mean (s.d.)*	n	*Mean (s.d.)*	n	*Mean (s.d.)*	n	*Mean (s.d.)*	n	*Mean (s.d.)*
*11 y/o (KS 2)*												
English mark	1524	61.72 (13.77)	1872	66.43 (12.82)	296	60.96 (13.09)	315	64.88 (13.14)	344	58.35 (14.01)	366	62.63 (13.59)
Maths mark	1528	73.56 (18.20)	1861	70.57 (17.71)	295	73.75 (18.09)	315	68.10 (18.53)	345	69.36 (18.77)	365	66.79 (18.34)
Science mark	1528	63.24 (10.04)	1861	63.24 (9.97)	298	69.36 (18.77)	315	61.69 (10.42)	347	61.47 (9.92)	369	60.85 (10.69)
												
*13 y/o (KS 3)*												
English mark	1314	48.05 (16.37)	1600	55.42 (14.75)	252	50.27 (15.24)	282	53.30 (14.76)	297	45.05 (15.55)	323	50.02 (15.08)
Maths mark	1320	88.75 (21.20)	1603	86.82 (20.60)	255	91.36 (20.00)	282	83.41 (22.26)	291	84.30 (20.24)	324	80.98 (20.32)
Science mark	1324	100.71 (21.66)	1607	101.49 (22.68)	255	103.60 (22.34)	281	100.40 (22.17)	299	99.93 (23.99)	323	99.12 (23.95)
												
		*Mode (range)*		*Mode (range)*		*Mode (range)*		*Mode (range)*		*Mode (range)*		*Mode (range)*
*16 y/o (KS 4)*												
GCSE English	1461	6.37 (1.33) (Grade C)	1788	6.87 (1.20) (Grade C)	285	6.39 (1.26) (Grade C)	313	6.70 (1.23) (Grade C)	328	6.09 (1.40) (Grade C)	355	6.44 (1.27) (Grade C)
GCSE Maths	1399	6.49 (1.53) (Grade C)	1766	6.54 (1.53) (Grade C)	278	6.66 (1.56) (Grade C)	309	6.31 (1.50) (Grade C)	318	6.10 (1.61) (Grade C)	352	6.14 (1.53) (Grade C)
GCSE Science	843	6.40 (1.43) (Grade C)	1070	6.46 (1.41) (Grade C)	202	6.55 (1.40) (Grade C)	181	6.17 (1.41) (Grade C)	202	6.16 (1.39) (Grade C)	217	6.12 (1.32) (Grade C)

Abbreviations: GCSE, General Certificate of Secondary Education; KS, Key Stage; y/o, years old.

At Key Stages 2 and 3, raw scores are converted to levels (level 1–8, with 8 being the highest). By the end of Key Stage 2, most pupils will have reached level 4, and at the end of Key Stage 3, most pupils will have reached levels 5 and 6.

**Table 3:** Weight status at 11 predicting English mark at 11, 13 and 16 years old in males

*Model*	n	R^*2*^ *change*	*Overweight*				*Obese*			
			B *(s.e.)*	*95% CI*	β	P*-value*	B *(s.e.)*	*95% CI*	β	P*-value*
*11 years old*										
Unadjusted	2164	0.008	−0.76 (0.87)	−2.47 to 0.95	−0.019	0.385	−3.37 (0.82)	−4.97 to −1.76	−0.089	<0.001
Fully adjusted	879	0.004	−1.22 (1.13)	−3.44 to 1.01	−0.031	0.284	−2.52 (1.15)	−4.78 to −0.26	−0.063	0.029
										
*13 years old*										
Unadjusted	1863	0.008	2.22 (1.11)	0.05 to 4.39	0.047	0.045	−3.00 (1.03)	−5.03 to −0.98	−0.068	0.004
Fully adjusted	641	0.001	−0.69 (1.57)	−3.78 to 2.39	−0.015	0.660	−1.59 (1.62)	−4.76 to 1.58	−0.035	0.325
										
*16 years old*										
Unadjusted	2074	0.006	0.02 (0.09)	−0.15 to 0.19	0.005	0.829	−0.28 (0.08)	−0.44 to −0.12	−0.077	0.001
Fully adjusted	571	0.001	−0.08 (0.12)	−0.31 to 0.16	−0.023	0.528	−0.08 (0.12)	−0.33 to 0.16	−0.025	0.508
Adjust for BMI at 16 years	550	0.000	−0.03 (0.13)	−0.29 to 0.23	−0.008	0.842	0.01 (0.16)	−0.31 to 0.32	0.003	0.955

Abbreviations: BMI, body mass index; CI, confidence; IQ, intelligence quotient; MVPA, moderate-to-vigorous physical activity.

*Note:* Healthy weight is the reference group, thus *β*-values show overweight compared with normal and obese compared with normal. *B*=unstandardised—so actual change in mark. *R*^2^ change shows the unique variance with the addition of the weight status variables into the model.

Fully adjusted model (model 9) adjusts for: age; birth weight; gestation; age of mother at delivery; oily fish intake during pregnancy; whether mother smoked during pregnancy; pubertal status; ethnicity, maternal education; maternal social class; average daily number of minutes spent in MVPA; depressive symptoms at 11; full-scale IQ at 8 years. At 16 years, additional model (model 10) includes adjustment for BMI *Z*-score at 16 years.

**Table 4:** Weight status at 11 predicting English mark at 11, 13 and 16 years old in females

*Model*	n	R^*2*^ *change*	*Overweight*				*Obese*			
			B *(s.e.)*	*95% CI*	β	P*-value*	B *(s.e.)*	*95% CI*	β	P*-value*
*11 years old*										
Unadjusted	2553	0.011	−1.55 (0.79)	−3.10 to −0.003	−0.039	0.049	−3.80 (0.74)	−5.25 to −2.35	−0.102	<0.001
Fully adjusted	1246	0.001	0.20 (0.91)	−1.59 to 1.98	0.005	0.830	−1.35 (0.93)	−3.17 to 0.48	−0.035	0.148^a^
										
*13 years old*										
Unadjusted	2205	0.017	−2.11 (0.96)	−3.99 to −0.24	−0.047	0.027	−5.40 (0.90)	−7.17 to −3.63	−0.128	<0.001
Fully adjusted	875	0.007	−2.18 (1.29)	−4.71 to 0.36	−0.050	0.093	−3.71 (1.36)	−6.38 to −1.04	−0.082	0.006
										
*16 years old*										
Unadjusted	2456	0.016	−0.17 (0.08)	−0.32 to –0.03	−0.047	0.020	−0.43 (0.07)	−0.57 to −0.29	−0.124	<0.001
Fully adjusted	829	0.005	−0.14 (0.10)	−0.33 to 0.04	−0.044	0.133	−0.26 (0.11)	−0.47 to −0.05	−0.072	0.017
Adjust for BMI at 16 years	800	0.004	−0.19 (0.11)	−0.40 to 0.02	−0.058	0.073	−0.27 (0.13)	−0.53 to −0.01	−0.073	0.045

Abbreviations: BMI, body mass index; CI, confidence; IQ, intelligence quotient; MVPA, moderate-to-vigorous physical activity.

a N.B. The *β*-value was highly significant in all previous models and only became nonsignificant with the addition of IQ—see Supplementary Information.

*Note*: Normal weight is the reference group so *β*-values show overweight compared with normal and obese compared with normal. *B*=unstandardised—so actual change in mark. *R*^2^ change shows the unique variance with the addition of the weight status variables into the model.

Fully adjusted model (model 9) adjusts for: age; birth weight; gestation; age of mother at delivery; oily fish intake during pregnancy; whether mother smoked during pregnancy; pubertal status; ethnicity, maternal education; maternal social class; average daily number of minutes spent in MVPA; depressive symptoms at 11; full-scale IQ at 8 years. At 16 years, additional model (model 10) includes adjustment for BMI *Z*-score at 16 years.

**Table 5:** Mediation analysis detailing bootstrapped estimates of indirect effect and 95% CIs

	*Overweight*				*Obese*			
	*Males*		*Females*		*Males*		*Females*	
	*Beta* *(s.e.)*	*95% CI*	*Beta* *(s.e.)*	*95% CI*	*Beta* *(s.e.)*	*95% CI*	*Beta* *(s.e.)*	*95% CI*
*Depressive symptoms*								
Total effect	−0.04 (0.12)		−0.16 (0.10)		−0.04 (0.13)		−0.22 (0.11)*	
Direct effect	−0.04 (0.12)		−0.15 (0.10)		−0.03 (0.13		−0.22 (0.11)*	
Indirect effect	0.001 (0.01)	−0.01 to 0.02	−0.008 (0.01)	−0.037 to 0.003	−0.01 (0.01)	−0.06 to 0.01	−0.007 (0.01)	−0.03 to 0.004
								
*IQ*								
Total effect	−0.06 (0.13)		−0.16 (0.11)		−0.11 (0.14)		−0.26 (0.12)*	
Direct effect	−0.04 (0.12)		−0.15 (0.10)		−0.03 (0.13)		−0.22 (0.11)*	
Indirect effect	−0.02 (0.06)	−0.12 to 0.11	−0.01 (0.05)	−0.11 to 0.10	−0.08 (0.06)	−0.21 to 0.05	−0.05 (0.06)	−0.15 to 0.07
								
*Age of menarche*								
Total effect	—		−0.02 (0.12)		—		−0.39 (0.15)*	
Direct effect	—		−0.02 (0.12)		—		−0.37 (0.15)*	
Indirect effect	—		−0.01 (0.01)	−0.04 to 0.003	—		−0.02 (0.02)	−0.07 to 0.001

Abbreviations: CI, confidence; IQ, intelligence quotient.

*Note*: *P<0.05. See Supplementary Figures 1 and 2 in Supplementary Material for full description of mediation analyses.

**Table 6:** Associations between change in weight status between 11 and 16 years old and English attainment at 16 years old (GCSE): stable healthy weight as reference group

	*Stable overweight/obese*		*Overweight from healthy*		*Obese from healthy*		*Obese from overweight*		*Healthy weight from overweight/obese*	
	*Beta*	*95% CI*	*Beta*	*95% CI*	*Beta*	*95% CI*	*Beta*	*95% CI*	*Beta*	*95% CI*
*Males*										
Unadjusted	−0.40***	−0.58 to −0.21	−0.25	−0.63 to 0.13	−0.34	−1.05 to 0.37	−0.54	−1.12 to 0.05	−0.10	−0.34 to 0.13
Fully adjusted	−0.07	−0.30 to 0.16	−0.09	−0.50 to 0.32	−0.20	−0.88 to 0.48	−0.23	−1.08 to 0.62	−0.04	−0.32 to 0.25
										
*Females*										
Unadjusted	−0.41***	−0.57 to −0.24	−0.09	−0.34 to 0.16	−0.03	−0.93 to 0.22	−0.80***	−1.16 to −0.43	−0.11	−0.35 to 0.14
Fully adjusted	−0.20*	−0.40 to −0.004	0.11	−0.17 to 0.38	−0.01	−0.56 to 0.53	−0.54*	−0.95 to −0.13	−0.10	−0.35 to 0.16

Abbreviations: CI, confidence interval; GCSE, General Certificate of Secondary Education.

*P<0.05; *** *P*<0.001.
