# Supplementary material for: Obesity impairs academic attainment in adolescence: findings from ALSPAC, a UK cohort
Source: Int J Obes (Lond). 2014 Apr 8;38(10):1335–42. doi: 10.1038/ijo.2014.40 (PMC4189379; doi:10.1038/ijo.2014.40)
Supplement: Supplementary Information [file ijo201440x1.doc]

**Supplementary material**

**Mediation analyses additional information**

In order to infer mediation, the strength of relationship between two variables (path c in Figure 1) is compared to a model which includes a potential mediating variable (Figure 2). Path c in Figure 1 denotes the total effect that X has on Y and path c’ in Figure 2 denotes the direct effect, that is, the effect of X on Y while controlling for M, the mediating variable. The indirect effect that X has on Y via M (i.e. ab) is calculated as c - c’. Complete mediation is deemed when path c’ is zero, that is, that X no longer effects Y when M is controlled for. When partial mediation is apparent, the c’ path from X to Y is less than the c path, but is still significantly greater than zero.

M

a

b

c’

c

X Y X Y

Figure 1. Illustration of an unmediated model

Figure 2. Illustration of a mediated model

**Associations between weight status and academic attainment in males**

**At 11 years old**

Supplementary table 1.Weight status at 11 predicting English mark at 11 in males

|  |  | | Overweight | | | Obese | | |
| --- | --- | --- | --- | --- | --- | --- | --- | --- |
| Model | n | R^2^ change | B (SE) | 95% CI | p -value | B | 95% CI | p -value |
| 1 | 2164 | 0.008 | -0.76 (0.87) | -2.47 to 0.95 | 0.39 | -3.37 (0.82) | -4.97 to -1.76 | <0.00 |
| 2 | 2164 | 0.008 | -0.76 (0.87) | -2.47 to 0.95 | 0.38 | -3.37 (0.82) | -4.97 to -1.76 | <0.00 |
| 3 | 2045 | 0.008 | -1.03 (0.90) | -2.79 to 0.73 | 0.25 | -3.43 (0.86) | -5.11 to -1.75 | <0.00 |
| 4 | 1933 | 0.006 | -0.38 (0.90) | -2.15 to 1.39 | 0.67 | -2.98 (0.86) | -4.67 to -1.29 | 0.001 |
| 5 | 1251 | 0.004 | -0.59 (1.12) | -2.78 to 1.61 | 0.60 | -2.47 (1.07) | -4.57 to -0.38 | 0.021 |
| 6 | 1131 | 0.002 | 0.27 (1.12) | -1.93 to 2.47 | 0.81 | -1.68 (1.08) | -3.80 to 0.44 | 0.120 |
| 7 | 968 | 0.007 | -0.44 (1.21) | -2.80 to 1.93 | 0.72 | -3.39 (1.22) | -5.78 to -1.00 | 0.006 |
| 8 | 952 | 0.006 | -0.42 (1.22) | -2.81 to 1.96 | 0.73 | -3.27 (1.23) | -5.68 to -0.87 | 0.008 |
| 9 | 879 | 0.004 | -1.22 (1.13) | -3.44 to 1.01 | 0.28 | -2.52 (1.15) | -4.78 to -0.26 | 0.029 |

Note: Healthy weight is the reference group so beta values show overweight and obese compared to participants who were a healthy weight. B = unstandardized so indicating the actual change in academic attainment. R^2^ change shows the unique variance with the addition of the weight status variables into the model. Model 1: unadjusted; model 2 adjusts for age; model 3 adjusts also birthweight and gestation; model 4 adjusts also age of mother at delivery, oily fish intake during pregnancy and whether mother smoked during pregnancy; model 5 also adjusts for pubertal status; model 6 also adjusts for ethnicity, maternal education and social class; model 7 also adjusts for MVPA; model 8 adds depressive symptoms at 11; model 9 adjust for all confounders in model 8 plus full scale IQ at 8.

Supplementary table 2.Weight status at 11 predicting Maths mark at 11 in males

|  |  | | Overweight | | | Obese | | |
| --- | --- | --- | --- | --- | --- | --- | --- | --- |
| Model | n | R^2^ change | B (SE) | 95% CI | p -value | B | 95% CI | p -value |
| 1 | 2168 | 0.007 | 0.20 (1.16) | -2.09 to 2.48 | 0.867 | -4.20 (1.09) | -6.33 to -2.06 | <0.001 |
| 2 | 2168 | 0.007 | 0.22 (1.16) | -2.06 to 2.50 | 0.849 | -4.17 (1.09) | -6.31 to -2.04 | <0.001 |
| 3 | 2049 | 0.007 | 0.04 (1.19) | -2.30 to 2.38 | 0.973 | -4.10 (1.14) | -6.33 to -1.88 | <0.001 |
| 4 | 1938 | 0.006 | 0.20 (1.21) | -2.17 to 2.57 | 0.866 | -3.92 (1.15) | -6.18 to -1.66 | 0.001 |
| 5 | 1253 | 0.003 | 0.22 (1.50) | -2.71 to 3.15 | 0.883 | -2.88 (1.43) | -5.69 to -0.08 | 0.044 |
| 6 | 1134 | 0.001 | 0.98 (1.52) | -2.00 to 3.96 | 0.520 | -1.50 (1.47) | -4.37 to 1.38 | 0.307 |
| 7 | 971 | 0.001 | 0.58 (1.63) | -2.62 to 3.78 | 0.722 | -1.77 (1.64) | -4.98 to 1.45 | 0.282 |
| 8 | 955 | 0.002 | 1.04 (1.62) | -2.15 to 4.22 | 0.523 | -1.57 (1.63) | -4.77 to 1.64 | 0.337 |
| 9 | 882 | 0.001 | 0.60 (1.44) | -2.23 to 3.42 | 0.679 | -1.33 (1.46) | -4.19 to 1.53 | 0.363 |

Note: Healthy weight is the reference group so beta values show overweight and obese compared to participants who were a healthy weight. B = unstandardized so indicating the actual change in academic attainment. R^2^ change shows the unique variance with the addition of the weight status variables into the model. Model 1: unadjusted; model 2 adjusts for age; model 3 adjusts also birthweight and gestation; model 4 adjusts also age of mother at delivery, oily fish intake during pregnancy and whether mother smoked during pregnancy; model 5 also adjusts for pubertal status; model 6 also adjusts for ethnicity, maternal education and social class; model 7 also adjusts for MVPA; model 8 adds depressive symptoms at 11; model 9 adjust for all confounders in model 8 plus full scale IQ at 8.

Supplementary table 3.Weight status at 11 predicting Science mark at 11 in males

|  |  | | Overweight | | | Obese | | |
| --- | --- | --- | --- | --- | --- | --- | --- | --- |
| Model | n | R^2^ change | B (SE) | 95% CI | p -value | B (SE) | 95% CI | p -value |
| 1 | 2173 | 0.004 | -0.05 (0.63) | -1.29 to 1.20 | 0.943 | -1.77 (0.60) | -2.94 to -0.61 | 0.003 |
| 2 | 2173 | 0.004 | -0.02 (0.63) | -1.26 to 1.22 | 0.979 | -1.75 (0.59) | -2.91 to -0.58 | 0.003 |
| 3 | 2054 | 0.004 | -0.28 (0.65) | -1.55 to 0.99 | 0.671 | -1.84 (0.62) | -3.05 to -0.62 | 0.003 |
| 4 | 1943 | 0.003 | 0.27 (0.66) | -1.03 to 1.56 | 0.688 | -1.55 (0.63) | -2.79 to -0.32 | 0.014 |
| 5 | 1256 | 0.001 | -0.51 (0.82) | -2.12 to 1.10 | 0.532 | -0.92 (0.79) | -2.46 to 0.62 | 0.241 |
| 6 | 1136 | 0.000 | 0.19 (0.83) | -1.43 to 1.82 | 0.815 | 0.07 (0.80) | -1.49 to 1.64 | 0.927 |
| 7 | 972 | 0.000 | 0.09 (0.88) | -1.64 to 1.82 | 0.921 | -0.36 (0.89) | -2.10 to 1.39 | 0.689 |
| 8 | 956 | 0.000 | 0.23 (0.88) | -1.49 to 1.96 | 0.792 | -0.27 (0.89) | -2.01 to 1.48 | 0.765 |
| 9 | 883 | 0.000 | -0.51 (0.77) | -2.02 to 1.00 | 0.508 | 0.14 (0.78) | -1.39 to 1.67 | 0.858 |

Note: Healthy weight is the reference group so beta values show overweight and obese compared to participants who were a healthy weight. B = unstandardized so indicating the actual change in academic attainment. R^2^ change shows the unique variance with the addition of the weight status variables into the model. Model 1: unadjusted; model 2 adjusts for age; model 3 adjusts also birthweight and gestation; model 4 adjusts also age of mother at delivery, oily fish intake during pregnancy and whether mother smoked during pregnancy; model 5 also adjusts for pubertal status; model 6 also adjusts for ethnicity, maternal education and social class; model 7 also adjusts for MVPA; model 8 adds depressive symptoms at 11; model 9 adjust for all confounders in model 8 plus full scale IQ at 8.

**At 13 years old**

Supplementary Table 4. Weight status at 11 predicting English mark at 13 in males

|  |  | | Overweight | | | Obese | | |
| --- | --- | --- | --- | --- | --- | --- | --- | --- |
| Model | n | R^2^ change | B (SE) | 95% CI | p -value | B | 95% CI | p -value |
| 1 | 1863 | 0.008 | 2.22 (1.11) | 0.05 to 4.39 | 0.045 | -3.00 (1.03) | -5.03 to -0.98 | 0.004 |
| 2 | 1593 | 0.010 | 1.79 (1.19) | -0.54 to 4.12 | 0.13 | -3.89 (1.11) | -6.07 to -1.70 | <0.001 |
| 3 | 1496 | 0.010 | 1.96 (1.22) | -0.44 to 4.36 | 0.11 | -3.55 (1.17) | -5.84 to -1.26 | 0.002 |
| 4 | 1423 | 0.009 | 2.63 (1.21) | 0.25 to 5.01 | 0.03 | -2.86 (1.16) | -5.13to -0.60 | 0.013 |
| 5 | 904 | 0.008 | 1.77 (1.51) | -1.20 to 4.74 | 0.24 | -3.30 (1.47) | -6.20 to -0.41 | 0.025 |
| 6 | 823 | 0.005 | 1.63 (1.53) | -1.38 to 4.63 | 0.29 | -2.34 (1.47) | -5.22 to 0.54 | 0.111 |
| 7 | 729 | 0.003 | 0.56 (1.59) | -2.55 to 3.68 | 0.72 | -2.34 (1.63) | -5.54 to 0.86 | 0.152 |
| 8 | 683 | 0.002 | 0.60 (1.64) | -2.62 to 3.81 | 0.716 | -1.90 (1.71) | -5.25 to 1.45 | 0.266 |
| 9 | 641 | 0.001 | -0.69 (1.57) | -3.78 to 2.39 | 0.66 | -1.59 (1.62) | -4.76 to 1.58 | 0.325 |

Model 1: unadjusted; model 2 adjust for age; model 3 adjust also birthweight and gestation; model 4 adjusts also age of mother at delivery, oily fish intake during pregnancy and whether mother smoked during pregnancy; model 5also adjusts for pubertal status; model 6also adjusts for ethnicity, maternal education and social class; model 7 also adjusts for MVPA; model 8 adjust for all confounders in model 7 but adds depressive symptoms at 11; Model 9 adjusts for all in model 8 and adds full scale IQ at 8.

Supplementary Table 5 Weight status at 11 predicting Maths mark at 13 in males

|  |  | | Overweight | | | Obese | | |
| --- | --- | --- | --- | --- | --- | --- | --- | --- |
| Model | n | R^2^ change | B (SE) | 95% CI | p -value | B | 95% CI | p -value |
| 1 | 1866 | 0.009 | 2.61 (1.43) | -0.20 to 5.41 | 0.068 | -4.46 (1.35) | -7.11 to -1.80 | 0.001 |
| 2 | 1597 | 0.009 | 1.93 (1.55) | -1.11 to 4.97 | 0.213 | -4.93 (1.46) | -7.80 to -2.06 | 0.001 |
| 3 | 1499 | 0.010 | 2.12 (1.60) | -1.02 to 5.25 | 0.186 | -5.08 (1.53) | -8.08 to -2.08 | 0.001 |
| 4 | 1426 | 0.009 | 2.48 (1.62) | -0.70 to 5.65 | 0.127 | -4.48 (1.55) | -7.52 to -1.44 | 0.004 |
| 5 | 906 | 0.012 | 3.90 (2.06) | -0.15 to 7.94 | 0.059 | -4.61 (2.00) | -8.55 to -0.68 | 0.022 |
| 6 | 823 | 0.008 | 3.37 (2.13) | -0.81 to 7.55 | 0.114 | -3.60 (2.05) | -7.63 to 0.43 | 0.080 |
| 7 | 729 | 0.007 | 2.03 (2.24) | -2.37 to 6.44 | 0.365 | -4.52 (2.31) | -9.06 to 0.03 | 0.051 |
| 8 | 684 | 0.007 | 2.49 (2.33) | -2.09 to 7.07 | 0.287 | -4.04 (2.43) | -8.81 to 0.74 | 0.097 |
| 9 | 642 | 0.003 | -0.05 (2.19) | -4.35 to 4.25 | 0.980 | -3.46 (2.25) | -7.87 to 0.96 | 0.125 |

Model 1: unadjusted; model 2 adjust for age; model 3 adjust also birthweight and gestation; model 4 adjusts also age of mother at delivery, oily fish intake during pregnancy and whether mother smoked during pregnancy; model 5also adjusts for pubertal status; model 6also adjusts for ethnicity, maternal education and social class; model 7 also adjusts for MVPA; model 8 adjust for all confounders in model 7 but adds depressive symptoms at 11; Model 9 adjusts for all in model 8 and adds full scale IQ at 8.

Supplementary Table 6 Weight status at 11 predicting Science mark at 13 in males

|  |  | | Overweight | | | Obese | | |
| --- | --- | --- | --- | --- | --- | --- | --- | --- |
| Model | n | R^2^ change | B (SE) | 95% CI | p -value | B | 95% CI | p -value |
| 1 | 1878 | 0.002 | 2.89 (1.51) | -0.08 to 5.86 | 0.057 | -0.79 (1.42) | -3.57 to 2.00 | 0.580 |
| 2 | 1605 | 0.001 | 1.48 (1.62) | -1.69 to 4.65 | 0.359 | -0.81 (1.51) | -3.78 to 2.16 | 0.592 |
| 3 | 1507 | 0.001 | 1.44 (1.67) | -1.82 to 4.71 | 0.386 | -0.48 (1.58) | -3.58 to 2.62 | 0.762 |
| 4 | 1433 | 0.002 | 2.56 (1.69) | -0.75 to 5.87 | 0.130 | 0.22 (1.60) | -2.93 to 3.36 | 0.892 |
| 5 | 909 | 0.003 | 3.41 (2.08) | -0.68 to 7.50 | 0.102 | -0.45 (2.02) | -4.41 to 3.51 | 0.823 |
| 6 | 826 | 0.004 | 3.99 (2.21) | -0.35 to 8.34 | 0.072 | 0.88 (2.12) | -3.28 to 5.05 | 0.677 |
| 7 | 731 | 0.005 | 4.32 (2.29) | -0.17 to 8.80 | 0.059 | 0.77 (2.34) | -3.83 to 5.36 | 0.743 |
| 8 | 686 | 0.005 | 4.60 (2.34) | -0.003 to 9.20 | 0.050 | 0.96 (2.42) | -3.79 to 5.71 | 0.691 |
| 9 | 645 | 0.002 | 2.62 (2.39) | -2.08 to 7.31 | 0.274 | 1.43 (2.43) | -3.34 to 6.20 | 0.556 |

Model 1: unadjusted; model 2 adjust for age; model 3 adjust also birthweight and gestation; model 4 adjusts also age of mother at delivery, oily fish intake during pregnancy and whether mother smoked during pregnancy; model 5 also adjusts for pubertal status; model 6 also adjusts for ethnicity, maternal education and social class; model 7 also adjusts for MVPA; model 8 adjust for all confounders in model 7 but adds depressive symptoms at 11; Model 9 adjusts for all in model 8 and adds full scale IQ at 8.

**At 16 years old**

Supplementary Table 7. Weight status at 11 predicting English mark at 16 in males

|  |  | | Overweight | | | Obese | | |
| --- | --- | --- | --- | --- | --- | --- | --- | --- |
| Model | n | R^2^ change | B (SE) | 95% CI | p -value | B | 95% CI | p -value |
| 1 | 2074 | 0.006 | 0.02 (0.09) | -0.15 to 0.19 | 0.829 | -0.28 (0.08) | -0.44 to -0.12 | 0.001 |
| 2 | 1549 | 0.012 | -0.15 (0.10) | -0.34 to 0.04 | 0.122 | -0.38 (0.09) | -0.56 to -0.21 | <0.001 |
| 3 | 1451 | 0.011 | -0.16 (0.10) | -0.35 to 0.04 | 0.116 | -0.37 (0.10) | -0.56 to -0.19 | <0.001 |
| 4 | 1379 | 0.008 | -0.08 (0.10) | -0.27 to 0.11 | 0.419 | -0.32 (0.09) | -0.50 to -0.14 | 0.001 |
| 5 | 811 | 0.007 | 0.13 (0.12) | -0.11 to 0.37 | 0.298 | -0.24 (0.12) | -0.47 to -0.002 | 0.048 |
| 6 | 738 | 0.004 | 0.09 (0.12) | -0.15 to 0.33 | 0.468 | -0.20 (0.12) | -0.43 to 0.03 | 0.092 |
| 7 | 656 | 0.004 | -0.03 (0.12) | -0.27 to 0.21 | 0.820 | -0.23 (0.13) | -0.48 to 0.01 | 0.063 |
| 8 | 614 | 0.004 | -0.06 (0.12) | -0.30 to 0.19 | 0.642 | -0.22 (0.13) | -0.48 to 0.03 | 0.088 |
| 9 | 571 | 0.001 | -0.08 (0.12) | -0.31 to 0.16 | 0.528 | -0.08 (0.12) | -0.33 to 0.16 | 0.508 |
| 10 | 550 | 0.000 | -0.03 (0.13) | -0.29 to 0.23 | 0.842 | 0.01(0.16) | -0.31 to 0.32 | 0.955 |

Model 1: unadjusted; model 2 adjust for age; model 3 adjust also birthweight and gestation; model 4 adjusts also age of mother at delivery, oily fish intake during pregnancy and whether mother smoked during pregnancy; model 5also adjusts for pubertal status; model 6also adjusts for ethnicity, maternal education and social class; model 7 also adjusts for MVPA; model 8 adjust for all confounders in model 7 but adds depressive symptoms at 11; Model 9 adjusts for all in model 8 and adds full scale IQ at 8. Model 10 includes adjustment for BMI z score at 16. NB beta values are smaller at 16 year old analysis but GCSE is on a 9 point scale rather than 100 point (as at for 11 and 13).

Supplementary Table 8 Weight status at 11 predicting Maths mark at 16 in males

|  |  | | Overweight | | | Obese | | |
| --- | --- | --- | --- | --- | --- | --- | --- | --- |
| Model | n | R^2^ change | B (SE) | 95% CI | p -value | B | 95% CI | p -value |
| 1 | 1995 | 0.011 | 0.17 (0.10) | -0.03 to 0.36 | 0.104 | -0.39 (0.10) | -0.58 to -0.20 | <0.001 |
| 2 | 1476 | 0.015 | 0.01 (0.11) | -0.21 to 0.23 | 0.911 | -0.50 (0.11) | -0.71 to -0.29 | <0.001 |
| 3 | 1380 | 0.013 | 0.004 (0.12) | -0.22 to 0.23 | 0.970 | -0.47 (0.11) | -0.68 to -0.25 | <0.001 |
| 4 | 1309 | 0.012 | 0.05 (0.11) | -0.17 to 0.27 | 0.667 | -0.43 (0.11) | -0.65 to -0.22 | <0.001 |
| 5 | 756 | 0.026 | 0.26 (0.14) | -0.02 to 0.55 | 0.066 | -0.54 (0.14) | -0.82 to -0.27 | <0.001 |
| 6 | 685 | 0.021 | 0.24 (0.15) | -0.05 to 0.53 | 0.098 | -0.49 (0.14) | -0.76 to -0.21 | 0.001 |
| 7 | 609 | 0.020 | 0.12 (0.15) | -0.17 to 0.41 | 0.423 | -0.54 (0.15) | -0.84 to -0.24 | <0.001 |
| 8 | 570 | 0.017 | 0.14 (0.15) | -0.16 to 0.44 | 0.355 | -0.50 (0.16) | -0.81 to -0.18 | 0.002 |
| 9 | 529 | 0.010 | 0.16 (0.14) | -0.12 to 0.43 | 0.255 | -0.37 (0.15) | -0.65 to -0.08 | 0.013 |
| 10 | 512 | 0.007 | 0.18 (0.15) | -0.12 to 0.48 | 0.237 | -0.29 (0.19) | -0.66 to 0.07 | 0.113 |

Model 1: unadjusted; model 2 adjust for age; model 3 adjust also birthweight and gestation; model 4 adjusts also age of mother at delivery, oily fish intake during pregnancy and whether mother smoked during pregnancy; model 5 also adjusts for pubertal status; model 6 also adjusts for ethnicity, maternal education and social class; model 7 also adjusts for MVPA; model 8 adjust for all confounders in model 7 but adds depressive symptoms at 11; Model 9 adjusts for all in model 8 and adds full scale IQ at 8; model 10 adjusts for BMI z score at 16 too.

Supplementary Table 9 Weight status at 11 predicting Science mark at 16 in males

|  |  | | Overweight | | | Obese | | |
| --- | --- | --- | --- | --- | --- | --- | --- | --- |
| Model | n | R^2^ change | B (SE) | 95% CI | p -value | B | 95% CI | p -value |
| 1 | 1228 | 0.006 | 0.15 (0.12) | -0.08 to 0.38 | 0.191 | -0.23 (0.11) | -0.45 to -0.01 | 0.037 |
| 2 | 878 | 0.007 | -0.09 (0.13) | -0.34 to 0.17 | 0.513 | -0.32 (0.13) | -0.56 to -0.07 | 0.012 |
| 3 | 841 | 0.006 | -0.08 (0.13) | -0.34 to 0.18 | 0.542 | -0.29 (0.13) | -0.55 to -0.03 | 0.027 |
| 4 | 801 | 0.005 | -0.06 (0.13) | -0.32 to 0.19 | 0.636 | -0.28 (0.13) | -0.52 to -0.03 | 0.029 |
| 5 | 451 | 0.011 | 0.31 (0.17) | -0.02 to 0.64 | 0.067 | -0.19 (0.17) | -0.51 to 0.14 | 0.263 |
| 6 | 402 | 0.007 | 0.26 (0.17) | -0.09 to 0.60 | 0.141 | -0.14 (0.17) | -0.47 to 0.18 | 0.389 |
| 7 | 363 | 0.009 | 0.08 (0.18) | -0.28 to 0.43 | 0.666 | -0.33 (0.18) | -0.67 to 0.02 | 0.066 |
| 8 | 341 | 0.011 | 0.13 (0.18) | -0.24 to 0.49 | 0.490 | -0.36 (0.18) | -0.72 to 0.003 | 0.052 |
| 9 | 319 | 0.004 | 0.08 (0.17) | -0.25 to 0.41 | 0.649 | -0.22 (0.17) | -0.54 to 0.11 | 0.194 |
| 10 | 308 | 0.005 | -0.01 (0.19) | -0.37 to 0.36 | 0.966 | -0.32 (0.21) | -0.73 to 0.10 | 0.139 |

Model 1: unadjusted; model 2 adjust for age; model 3 adjust also birthweight and gestation; model 4 adjusts also age of mother at delivery, oily fish intake during pregnancy and whether mother smoked during pregnancy; model 5 also adjusts for pubertal status; model 6 also adjusts for ethnicity, maternal education and social class; model 7 also adjusts for MVPA; model 8 adjust for all confounders in model 7 but adds depressive symptoms at 11; Model 9 adjusts for all in model 8 and adds full scale IQ at 8; model 10 adjusts for BMI z score at 16 too.

**Associations between weight status and academic attainment in females**

**At 11 years old**

Supplementary Table 10. Weight status at 11 predicting English mark at 11 in females

|  |  | | Overweight | | | Obese | | |
| --- | --- | --- | --- | --- | --- | --- | --- | --- |
| Model | n | R^2^ change | B (SE) | 95% CI | p -value | B | 95% CI | p -value |
| 1 | 2553 | 0.011 | -1.55 (0.79) | -3.10 to -0.003 | 0.049 | -3.80 (0.74) | -5.25 to -2.35 | <0.001 |
| 2 | 2553 | 0.011 | -1.55 (0.79) | -3.10 to -0.002 | 0.050 | -3.80 (0.74) | -5.25 to -2.35 | <0.001 |
| 3 | 2396 | 0.010 | -1.84 (0.82) | -3.45 to -0.24 | 0.024 | -3.67 (0.77) | -5.18 to -2.17 | <0.001 |
| 4 | 2265 | 0.006 | -1.49 (0.83) | -3.10 to 0.13 | 0.072 | -2.90 (0.79) | -4.44 to -1.35 | <0.001 |
| 5 | 1797 | 0.010 | -1.47 (0.95) | -3.34 to 0.40 | 0.123 | -3.89 (0.90) | -5.66 to -2.12 | <0.001 |
| 6 | 1591 | 0.006 | 0.21 (0.95) | -1.66 to 2.07 | 0.830 | -2.86 (0.93) | -4.68 to -1.04 | 0.002 |
| 7 | 1378 | 0.004 | 0.23 (1.02) | -1.77 to 2.23 | 0.821 | -2.38 (1.02) | -4.37 to -0.38 | 0.020 |
| 8 | 1356 | 0.003 | 0.09 (1.03) | -1.93 to 2.11 | 0.930 | -2.30 (1.03) | -4.32 to -0.29 | 0.025 |
| 9 | 1246 | 0.001 | 0.20 (0.91) | -1.59 to 1.98 | 0.830 | -1.35 (0.93) | -3.17 to 0.48 | 0.148 |

Note: Healthy weight is the reference group so beta values show overweight and obese compared to participants who were a healthy weight. B = unstandardized so indicating the actual change in academic attainment. R^2^ change shows the unique variance with the addition of the weight status variables into the model. Model 1: unadjusted; model 2 adjusts for age; model 3 adjusts also birthweight and gestation; model 4 adjusts also age of mother at delivery, oily fish intake during pregnancy and whether mother smoked during pregnancy; model 5 also adjusts for pubertal status; model 6 also adjusts for ethnicity, maternal education and social class; model 7 also adjusts for MVPA; model 8 adds depressive symptoms at 11; model 9 adjust for all confounders in model 8 plus full scale IQ at 8.

Supplementary Table 11. Weight status at 11 predicting Maths mark at 11 in females

|  |  | | Overweight | | | Obese | | |
| --- | --- | --- | --- | --- | --- | --- | --- | --- |
| Model | n | R^2^ change | B (SE) | 95% CI | p -value | B | 95% CI | p -value |
| 1 | 2541 | 0.006 | -2.47 (1.09) | -4.61 to -0.33 | 0.024 | -3.78 (1.03) | -5.79 to -1.77 | <0.001 |
| 2 | 2541 | 0.006 | -2.48 (1.09) | -4.62 to -0.34 | 0.023 | -3.77 (1.03) | -5.78 to -1.76 | <0.001 |
| 3 | 2386 | 0.008 | -3.03 (1.13) | -5.25 to -0.81 | 0.007 | -4.00 (1.06) | -6.08 to -1.92 | <0.001 |
| 4 | 2257 | 0.005 | -3.10 (1.15) | -5.35 to -0.85 | 0.007 | -2.93 (1.10) | -5.08 to -0.77 | 0.008 |
| 5 | 1788 | 0.007 | -3.21 (1.31) | -5.78 to -0.64 | 0.014 | -3.77 (1.25) | -6.22 to -1.31 | 0.003 |
| 6 | 1582 | 0.001 | -1.24 (1.31) | -3.82 to 1.33 | 0.345 | -1.65 (1.29) | -4.18 to 0.87 | 0.198 |
| 7 | 1372 | 0.001 | -1.39 (1.38) | -4.11 to 1.32 | 0.314 | -1.64 (1.39) | -4.36 to 1.08 | 0.237 |
| 8 | 1350 | 0.001 | -1.56 (1.39) | -4.28 to 1.17 | 0.262 | -1.55 (1.39) | -4.27 to 1.18 | 0.266 |
| 9 | 1240 | 0.001 | -1.67 (1.20) | -4.03 to 0.69 | 0.165 | -0.87 (1.24) | -3.30 to 1.56 | 0.482 |

Note: Healthy weight is the reference group so beta values show overweight and obese compared to participants who were a healthy weight. B = unstandardized so indicating the actual change in academic attainment. R^2^ change shows the unique variance with the addition of the weight status variables into the model. Model 1: unadjusted; model 2 adjusts for age; model 3 adjusts also birthweight and gestation; model 4 adjusts also age of mother at delivery, oily fish intake during pregnancy and whether mother smoked during pregnancy; model 5 also adjusts for pubertal status; model 6 also adjusts for ethnicity, maternal education and social class; model 7 also adjusts for MVPA; model 8 adds depressive symptoms at 11; model 9 adjust for all confounders in model 8 plus full scale IQ at 8.

Supplementary Table 12. Weight status at 11 predicting Science mark at 11 in females

|  |  | | Overweight | | | Obese | | |
| --- | --- | --- | --- | --- | --- | --- | --- | --- |
| Model | n | R^2^ change | B (SE) | 95% CI | p -value | B | 95% CI | p -value |
| 1 | 2546 | 0.008 | -1.55 (0.62) | -2.76 to -0.34 | 0.012 | -2.39 (0.58) | -3.52 to -1.25 | <0.001 |
| 2 | 2546 | 0.008 | -1.59 (0.62) | -2.80 to -0.38 | 0.010 | -2.35 (0.58) | -3.48 to -1.22 | <0.001 |
| 3 | 2389 | 0.008 | -1.75 (0.64) | -3.00 to -0.50 | 0.006 | -2.31 (0.59) | -3.47 to -1.14 | <0.001 |
| 4 | 2259 | 0.005 | -1.60 (0.64) | -2.85 to -0.34 | 0.013 | -1.72 (0.61) | -2.91 to -0.52 | 0.005 |
| 5 | 1787 | 0.009 | -1.45 (0.73) | -2.89 to -0.01 | 0.048 | -2.61 (0.70) | -3.98 to -1.25 | <0.001 |
| 6 | 1582 | 0.003 | -0.01 (0.73) | -1.45 to 1.43 | 0.988 | -1.74 (0.72) | -3.14 to -0.33 | 0.015 |
| 7 | 1374 | 0.003 | -0.28 (0.79) | -1.81 to 1.26 | 0.726 | -1.59 (0.78) | -3.12 to -0.05 | 0.043 |
| 8 | 1352 | 0.003 | -0.32 (0.79) | -1.88 to 1.23 | 0.682 | -1.58 (0.79) | -3.13 to -0.03 | 0.045 |
| 9 | 1242 | 0.001 | -0.47 (0.68) | -1.81 to 0.86 | 0.485 | -1.00 (0.70) | -2.36 to 0.37 | 0.152 |

Note: Healthy weight is the reference group so beta values show overweight and obese compared to participants who were a healthy weight. B = unstandardized so indicating the actual change in academic attainment. R^2^ change shows the unique variance with the addition of the weight status variables into the model. Model 1: unadjusted; model 2 adjusts for age; model 3 adjusts also birthweight and gestation; model 4 adjusts also age of mother at delivery, oily fish intake during pregnancy and whether mother smoked during pregnancy; model 5 also adjusts for pubertal status; model 6 also adjusts for ethnicity, maternal education and social class; model 7 also adjusts for MVPA; model 8 adds depressive symptoms at 11; model 9 adjust for all confounders in model 8 plus full scale IQ at 8.

**At 13 years old**

Supplementary Table 13.Weight status at 11 predicting English mark at 13 in females

|  |  | | Overweight | | | Obese | | |
| --- | --- | --- | --- | --- | --- | --- | --- | --- |
| Model | n | R^2^ change | B (SE) | 95% CI | p -value | B | 95% CI | p -value |
| 1 | 2205 | 0.017 | -2.11 (0.96) | -3.99 to -0.24 | 0.027 | -5.40 (0.90) | -7.17 to -3.63 | <0.001 |
| 2 | 1862 | 0.014 | -1.80 (1.04) | -3.84 to 0.24 | 0.084 | -5.06 (1.00) | -7.01 to -3.11 | <0.001 |
| 3 | 1747 | 0.015 | -1.90 (1.07) | -4.01 to 0.20 | 0.076 | -5.26 (1.03) | -7.28 to -3.25 | <0.001 |
| 4 | 1655 | 0.011 | -1.71 (1.09) | -3.84 to 0.42 | 0.115 | -4.47 (1.06) | -6.54 to -2.40 | <0.001 |
| 5 | 1268 | 0.017 | -2.81 (1.27) | -5.30 to -0.31 | 0.028 | -5.70 (1.26) | -8.17 to -3.22 | <0.001 |
| 6 | 1127 | 0.011 | -1.38 (1.30) | -3.93 to 1.18 | 0.292 | -5.05 (1.33) | -7.66 to -2.43 | <0.001 |
| 7 | 1008 | 0.011 | -1.61 (1.40) | -4.36 to 1.13 | 0.249 | -5.00 (1.43) | -7.81 to -2.19 | 0.001 |
| 8 | 937 | 0.013 | -2.37 (1.44) | -5.20 to 0.45 | 0.100 | -5.35 (1.50) | -8.30 to -2.40 | <0.001 |
| 9 | 875 | 0.007 | -2.18 (1.29) | -4.71 to 0.36 | 0.093 | -3.71 (1.36) | -6.38 to -1.04 | 0.006 |

Model 1: unadjusted; model 2 adjust for age; model 3 adjust also birthweight and gestation; model 4 adjusts also age of mother at delivery, oily fish intake during pregnancy and whether mother smoked during pregnancy; model 5also adjusts for pubertal status; model 6also adjusts for ethnicity, maternal education and social class; model 7 also adjusts for MVPA; model 8 adjust for all confounders in model 7 but adds depressive symptoms at 11; Model 9 adjusts for all in model 8 and adds full scale IQ at 8.

Supplementary Table 14.Weight status at 11 predicting Maths mark at 13 in females

|  |  | | Overweight | | | Obese | | |
| --- | --- | --- | --- | --- | --- | --- | --- | --- |
| Model | n | R^2^ change | B (SE) | 95% CI | p -value | B | 95% CI | p -value |
| 1 | 2209 | 0.011 | -3.41 (1.34) | -6.04 to -0.78 | 0.011 | -5.85 (1.27) | -8.33 to -3.37 | <0.001 |
| 2 | 1864 | 0.012 | -3.13 (1.46) | -6.00 to -0.26 | 0.033 | -6.37 (1.41) | -9.13 to -3.61 | <0.001 |
| 3 | 1748 | 0.013 | -3.24 (1.52) | -6.22 to -0.26 | 0.033 | -6.61 (1.46) | -9.47 to -3.76 | <0.001 |
| 4 | 1654 | 0.010 | -2.71 (1.52) | -5.70 to 0.27 | 0.075 | -5.98 (1.48) | -8.89 to -3.08 | <0.001 |
| 5 | 1269 | 0.016 | -4.97 (1.78) | -8.45 to -1.48 | 0.005 | -7.41 (1.76) | -10.87 to -3.95 | <0.001 |
| 6 | 1128 | 0.011 | -4.10 (1.85) | -7.73 to -0.47 | 0.027 | -6.24 (1.89) | -9.95 to -2.53 | 0.001 |
| 7 | 1008 | 0.007 | -3.40 (1.98) | -7.29 to 0.49 | 0.086 | -4.88 (2.04) | -8.88 to -0.87 | 0.017 |
| 8 | 939 | 0.007 | -4.31 (2.04) | -8.32 to -0.30 | 0.035 | -4.53 (2.15) | -8.74 to -0.31 | 0.035 |
| 9 | 877 | 0.006 | -3.74 (1.98) | -7.62 to 0.15 | 0.060 | -4.38 (2.10) | -8.50 to -0.26 | 0.037 |

Model 1: unadjusted; model 2 adjust for age; model 3 adjust also birthweight and gestation; model 4 adjusts also age of mother at delivery, oily fish intake during pregnancy and whether mother smoked during pregnancy; model 5 also adjusts for pubertal status; model 6 also adjusts for ethnicity, maternal education and social class; model 7 also adjusts for MVPA; model 8 adjust for all confounders in model 7 but adds depressive symptoms at 11; Model 9 adjusts for all in model 8 and adds full scale IQ at 8.

Supplementary Table 15.Weight status at 11 predicting Science mark at 13 in females

|  |  | | Overweight | | | Obese | | |
| --- | --- | --- | --- | --- | --- | --- | --- | --- |
| Model | n | R^2^ change | B (SE) | 95% CI | p -value | B | 95% CI | p -value |
| 1 | 2211 | 0.001 | -1.09 (1.48) | -3.98 to 1.80 | 0.460 | -2.37 (1.39) | -5.10 to 0.36 | 0.088 |
| 2 | 1867 | 0.000 | 0.13 (1.61) | -3.04 to 3.29 | 0.937 | -1.33 (1.55) | -4.36 to 1.71 | 0.391 |
| 3 | 1750 | 0.000 | -0.27 (1.68) | -3.57 to 3.03) | 0.872 | -1.06 (1.61) | -4.22 to 2.09 | 0.510 |
| 4 | 1657 | 0.001 | 0.51 (1.72) | -2.87 to 3.88 | 0.769 | -1.36 (1.67) | -4.64 to 1.93 | 0.418 |
| 5 | 1270 | 0.001 | -1.66 (2.04) | -5.66 to 2.35 | 0.417 | -1.03 (2.03) | -5.01 to 2.94 | 0.610 |
| 6 | 1128 | 0.001 | -2.01 (2.14) | -6.21 to 2.20 | 0.350 | -0.65 (2.19) | -4.95 to 3.65 | 0.766 |
| 7 | 1008 | 0.001 | -1.43 (2.28) | -5.91 to 3.04 | 0.529 | 0.81 (2.34) | -3.79 to 5.40 | 0.731 |
| 8 | 938 | 0.001 | -2.32 (2.34) | -6.91 to 2.27 | 0.321 | 0.58 (2.45) | -4.23 to 5.39 | 0.814 |
| 9 | 879 | 0.001 | -1.20 (2.36) | -5.83 to 3.44 | 0.613 | 0.93 (2.50) | -3.96 to 5.83 | 0.708 |

Model 1: unadjusted; model 2 adjust for age; model 3 adjust also birthweight and gestation; model 4 adjusts also age of mother at delivery, oily fish intake during pregnancy and whether mother smoked during pregnancy; model 5 also adjusts for pubertal status; model 6 also adjusts for ethnicity, maternal education and social class; model 7 also adjusts for MVPA; model 8 adjust for all confounders in model 7 but adds depressive symptoms at 11; Model 9 adjusts for all in model 8 and adds full scale IQ at 8.

**At 16 years old**

Supplementary Table 16. Weight status at 11 predicting English mark at 16 in females

|  |  | | Overweight | | | Obese | | |
| --- | --- | --- | --- | --- | --- | --- | --- | --- |
| Model | n | R^2^ change | B (SE) | 95% CI | p -value | B | 95% CI | p -value |
| 1 | 2456 | 0.016 | -0.17 (0.08) | -0.32 to -0.03 | 0.020 | -0.43 (0.07) | -0.57 to -0.29 | <0.001 |
| 2 | 1883 | 0.016 | -0.14 (0.09) | -0.31 to 0.03 | 0.100 | -0.44 (0.08) | -0.60 to -0.28 | <0.001 |
| 3 | 1768 | 0.015 | -0.15 (0.09) | -0.33 to -0.02 | 0.084 | -0.44 (0.09) | -0.60 to -0.27 | <0.001 |
| 4 | 1678 | 0.010 | -0.10 (0.09) | -0.27 to 0.07 | 0.244 | -0.35 (0.08) | -0.51 to -0.18 | <0.001 |
| 5 | 1194 | 0.014 | -0.16 (0.10) | -0.35 to 0.04 | 0.108 | -0.42 (0.10) | -0.62 to -0.22 | <0.001 |
| 6 | 1069 | 0.011 | -0.12 (0.10) | -0.31 to 0.07 | 0.224 | -0.40 (0.11) | -0.60 to -0.19 | <0.001 |
| 7 | 944 | 0.011 | -0.16 (0.11) | -0.36 to 0.05 | 0.139 | -0.40 (0.11) | -0.62 to -0.17 | 0.001 |
| 8 | 878 | 0.011 | -0.15 (0.11) | -0.37 to 0.06 | 0.159 | -0.39 (0.12) | -0.62 to -0.16 | 0.001 |
| 9 | 829 | 0.005 | -0.14 (0.10) | -0.33 to 0.04 | 0.133 | -0.26 (0.11) | -0.47 to -0.05 | 0.017 |
| 10 | 800 | 0.004 | -0.19 (0.11) | -0.40 to 0.02 | 0.073 | -0.27 (0.13) | -0.53 to -0.01 | 0.045 |

Model 1: unadjusted; model 2 adjust for age; model 3 adjust also birthweight and gestation; model 4 adjusts also age of mother at delivery, oily fish intake during pregnancy and whether mother smoked during pregnancy; model 5also adjusts for pubertal status; model 6also adjusts for ethnicity, maternal education and social class; model 7 also adjusts for MVPA; model 8b adjust for all confounders in model 7 but adds depressive symptoms at 11; Model 9 adjusts for all in model 8 and adds full scale IQ at 8; model 10 adjusts for BMI z score at 16 too.

Supplementary Table 17. Weight status at 11 predicting Maths mark at 16 in females

|  |  | | Overweight | | | Obese | | |
| --- | --- | --- | --- | --- | --- | --- | --- | --- |
| Model | n | R^2^ change | B (SE) | 95% CI | p -value | B | 95% CI | p -value |
| 1 | 2427 | 0.009 | -0.23 (0.09) | -0.41 to -0.04 | 0.016 | -0.40 (0.09) | -0.57 to -0.22 | <0.001 |
| 2 | 1858 | 0.006 | -0.14 (0.11) | -0.35 to 0.07 | 0.183 | -0.32 (0.10) | -0.52 to -0.12 | 0.002 |
| 3 | 1744 | 0.006 | -0.17 (0.11) | -0.39 to 0.05 | 0.129 | -0.33 (0.11) | -0.54 to -0.12 | 0.002 |
| 4 | 1654 | 0.003 | -0.12 (0.11) | -0.33 to 0.10 | 0.293 | -0.25 (0.11) | -0.46 to -0.04 | 0.018 |
| 5 | 1177 | 0.008 | -0.23 (0.13) | -0.48 to 0.03 | 0.081 | -0.40 (0.14) | -0.66 to -0.13 | 0.003 |
| 6 | 1054 | 0.005 | -0.15 (0.13) | -0.40 to 0.10 | 0.244 | -0.33 (0.14) | -0.59 to -0.06 | 0.018 |
| 7 | 930 | 0.003 | -0.10 (0.14) | -0.36 to 0.17 | 0.474 | -0.28 (0.15) | -0.56 to 0.01 | 0.059 |
| 8 | 866 | 0.003 | -0.08 (0.14) | -0.35 to 0.19 | 0.567 | -0.27 (0.15) | -0.56 to 0.03 | 0.074 |
| 9 | 818 | 0.001 | -0.07 (0.12) | -0.31 to 0.17 | 0.551 | -0.15 (0.14) | -0.42 to 0.12 | 0.278 |
| 10 | 789 | 0.000 | -0.04 (0.13) | -0.31 to 0.22 | 0.743 | -0.13 (0.17) | -0.45 to 0.20 | 0.450 |

Model 1: unadjusted; model 2 adjust for age; model 3 adjust also birthweight and gestation; model 4 adjusts also age of mother at delivery, oily fish intake during pregnancy and whether mother smoked during pregnancy; model 5 also adjusts for pubertal status; model 6 also adjusts for ethnicity, maternal education and social class; model 7 also adjusts for MVPA; model 8 adjust for all confounders in model 7 but adds depressive symptoms at 11; Model 9 adjusts for all in model 8 and adds full scale IQ at 8; model 10 adjusts for BMI z score at 16 too.

Supplementary Table 18. Weight status at 11 predicting Science mark at 16 in females

|  |  | | Overweight | | | Obese | | |
| --- | --- | --- | --- | --- | --- | --- | --- | --- |
| Model | n | R^2^ change | B (SE) | 95% CI | p -value | B | 95% CI | p -value |
| 1 | 1468 | 0.010 | -0.29 (0.11) | -0.51 to -0.07 | 0.010 | -0.34 (0.10) | -0.55 to -0.14 | 0.001 |
| 2 | 1096 | 0.007 | -0.19 (0.13) | -0.44 to 0.07 | 0.145 | -0.32 (0.12) | -0.55 to -0.08 | 0.008 |
| 3 | 1039 | 0.007 | -0.18 (0.13) | -0.44 to 0.09 | 0.189 | -0.31 (0.12) | -0.55 to -0.06 | 0.014 |
| 4 | 982 | 0.003 | -0.13 (0.13) | -0.39 to 0.13 | 0.328 | -0.22 (0.13) | -0.46 to 0.03 | 0.083 |
| 5 | 685 | 0.007 | -0.18 (0.15) | -0.49 to 0.12 | 0.239 | -0.33 (0.15) | -0.63 to -0.03 | 0.031 |
| 6 | 607 | 0.007 | -0.13 (0.15) | -0.43 to 0.17 | 0.400 | -0.36 (0.16) | -0.68 to -0.03 | 0.031 |
| 7 | 531 | 0.007 | -0.14 (0.17) | -0.47 to 0.19 | 0.396 | -0.37 (0.18) | -0.72 to -0.02 | 0.038 |
| 8 | 492 | 0.007 | -0.11 (0.18) | -0.46 to 0.23 | 0.526 | -0.37 (0.19) | -0.74 to 0.001 | 0.051 |
| 9 | 465 | 0.001 | -0.12 (0.15) | -0.40 to 0.17 | 0.424 | -0.10 (0.16) | -0.42 to 0.22 | 0.533 |
| 10 | 442 | 0.001 | -0.14 (0.16) | -0.46 to 0.18 | 0.385 | -0.08 (0.21) | -0.50 to 0.33 | 0.694 |

Model 1: unadjusted; model 2 adjust for age; model 3 adjust also birthweight and gestation; model 4 adjusts also age of mother at delivery, oily fish intake during pregnancy and whether mother smoked during pregnancy; model 5 also adjusts for pubertal status; model 6 also adjusts for ethnicity, maternal education and social class; model 7 also adjusts for MVPA; model 8 adjust for all confounders in model 7 but adds depressive symptoms at 11; Model 9 adjusts for all in model 8 and adds full scale IQ at 8; model 10 adjusts for BMI z score at 16 too.

**Analyses with only complete data**

In order to ensure effects sizes in the fully adjusted models were due to confounding rather than missing data, for each association, model 1 was repeated for participants who had complete data at model 9. The resulting regression coefficients were slightly larger than when all available data were included and results summarised in supplementary tables 10 and 11.

Supplementary Table 19. Model 1 repeated with only participants who have complete data at model 9 for males

| Model |  | Overweight |  |  | Obese |  |
| --- | --- | --- | --- | --- | --- | --- |
|  | B (SE) | 95% CI | p -value | B (SE) | 95% CI | p -value |
| English at 11 years (n=879) | -0.65 (1.36) | -3.32 to 2.01 | 0.630 | -3.45 (1.36) | -6.11 to -0.79 | 0.011 |
| Maths at 11 years (n=882) | 1.10 (1.77) | -2.37 to 4.56 | 0.536 | -2.78 (1.76) | -6.23 to 0.67 | 0.114 |
| Science at 11 years (n=883) | -0.32 (0.96) | -2.20 to 1.55 | 0.735 | -1.18 (0.96) | -3.06 to 0.69 | 0.216 |
| English at 13 years (n=641) | 0.41 (1.81) | -3.14 to 3.96 | 0.822 | -3.84 (1.79) | -7.36 to -0.33 | 0.032 |
| Maths at 13 years (n=642) | 3.08 (2.51) | -1.85 to 8.00 | 0.221 | -4.07 (2.50) | -8.97 to 0.84 | 0.104 |
| Science at 13 years (n=645) | 3.49 (2.39) | -1.21 to 8.19 | 0.145 | 0.76 (2.35) | -3.85 to 5.38 | 0.746 |
| English at 16 years (n=571) | -0.07 (0.14) | -0.35 to 0.21 | 0.611 | -0.30 (0.14) | -0.58 to -0.03 | 0.032 |
| Maths at 16 years (n=529) | 0.22 (0.17) | -0.12 to 0.55 | 0.207 | -0.60 (0.17) | -0.94 to -0.27 | <0.001 |
| Science at 16 years (n=319) | 0.15 (0.22) | -0.27 to 0.58 | 0.480 | -0.39 (0.21) | -0.79 to 0.02 | 0.062 |

Supplementary Table 20. Model 1 repeated with only participants who have complete data at model 9 for females

| Model |  | Overweight |  |  | Obese |  |
| --- | --- | --- | --- | --- | --- | --- |
|  | B (SE) | 95% CI | p -value | B (SE) | 95% CI | p -value |
| English at 11 years (n=1247) | -1.26 (1.09) | -3.40 to 0.89 | 0.251 | -2.95 (1.10) | -5.09 to -0.80 | 0.007 |
| Maths at 11 years (n=1241) | -2.91 (1.47) | -5.82 to -0.05 | 0.046 | -2.78 (1.48) | -5.69 to 0.13 | 0.061 |
| Science at 11 years (n=1243) | -1.39 (0.83) | -3.03 to 0.25 | 0.096 | -2.39 (0.84) | -4.04 to -0.75 | 0.004 |
| English at 13 years (n=875) | -2.66 (1.49) | -5.59 to 0.27 | 0.075 | -4.74 (1.54) | -7.77 to -1.71 | 0.002 |
| Maths at 13 years (n=877) | -4.18 (2.14) | -8.37 to 0.02 | 0.051 | -6.14 (2.23) | -10.51 to -1.76 | 0.006 |
| Science at 13 years (n=879) | -1.78 (2.35) | -6.40 to 2.84 | 0.450 | -0.93 (2.45) | -5.73 to 3.87 | 0.704 |
| English at 16 years (n=829) | -0.16 (0.11) | -0.38 to 0.06 | 0.161 | -0.27 (0.13) | -0.52 to -0.03 | 0.030 |
| Maths at 16 years (n=818) | -0.12 (0.15) | -0.41 to 0.17 | 0.418 | -0.20 (0.16) | -0.52 to 0.12 | 0.210 |
| Science at 16 years (n=465) | -0.18 (0.18) | -0.53 to 0.18 | 0.329 | -0.15 (0.20) | -0.54 to 0.24 | 0.458 |
